# A cell wall-localized glycine-rich protein of dodder acts as pathogen-associated molecular pattern

**DOI:** 10.1080/19420889.2021.1918369

**Published:** 2021-05-03

**Authors:** Peter Slaby, Max Körner, Markus Albert

**Affiliations:** Department of Biology, Molecular Plant Physiology, Erlangen, Germany

**Keywords:** Glycine-rich protein, GRP, parasitic plants, *Cuscuta reflexa*, CuRe1

## Abstract

*Cuscuta reflexa* (giant dodder) is an obligate stem holoparasite withdrawing water, nutrients, and carbohydrates from its hosts. For a broad spectrum of host plants, *C. reflexa* usually stays unrecognized. The cultivated tomato *Solanum lycopersicum*, as one notable exception, possesses a leucine-rich repeat receptor protein (LRR-RP), Cuscuta receptor 1 (CuRe1), which enables tomato to recognize *C. reflexa* as a dangerous parasitic invader and to respond with plant immune responses. During the infection process, a glycine-rich protein (GRP) is freed from *C. reflexa* and gets detected by CuRe1. Here, we focus on the subcellular localization of the GRP within plant cell walls using a fluorescence based co-localization.

Parasitic plants partially or completely lost their photoautotrophic lifestyle and rely on host plants as nutrient sources. With specific organs, called haustoria, they penetrate host tissues and connect to the vasculature, withdrawing water and nutrients, and in case of holoparasitism also carbohydrates. Such infections cause tremendous damage to host plants and parasitic plants indeed cause crop losses of about 10 billion USD annually. In most cases, such an attack goes unrecognized by the host plant and the plant immune system is unable to reject the parasite (see Figure 1a).

**Figure 1.**
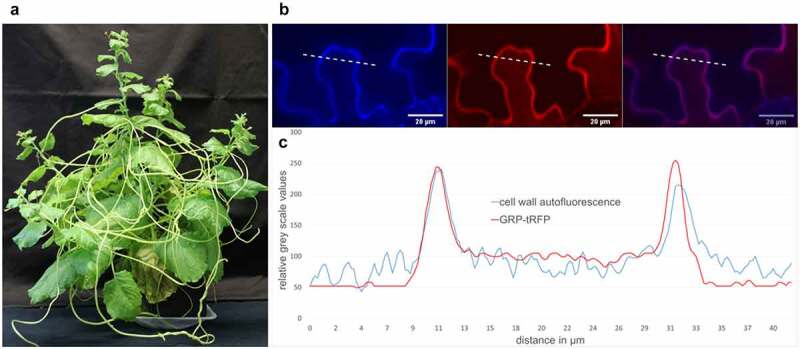
*Cuscutareflexa*and the localization of CrGRP. (a)*C.reflexa* growing on the susceptible host plant *Nicotiana benthamiana* (b) CrGRP-tagRFP was transiently overexpressed in *N.benthamiana*. RFP was excited at 561 nm and lignin auto fluorescence of the cell wall was excited at 405 nm with Zeiss confocal laser scanning microscope (LSM880, Carl Zeiss Microscopy GmbH, Carl-Zeiss-Promenade 10, 07745 Jena, Germany). Shown is the fluorescence in single channels (left + middle) and as overlay (right). (c) Overlay of GRP-tRFP fluorescence (red line) and cell wall autofluorescence (blue line) corresponding to dotted line in (b); fluorescence intensity is given in relative gray scale values; x-axis indicates distance in µm

*Cuscuta* spp. are rootless holoparasites with a very broad host spectrum and grow as vines on shoots of nearly all dicotyledonous hosts. In compatible interactions, the *Cuscuta* haustorium penetrates the host stem-tissues and connects to both host phloem and xylem. This procedure also includes the formation of chimeric cell walls as well as the development of interspecific plasmodesmata [1]. During the interaction of *Cuscuta reflexa* (giant dodder) with the resistant host plant *Solanum lycopersicum* (cultivated tomato), defense reactions are initiated to fend off the parasite. These defense reactions are visible on the tomato stem at the attempted haustorium penetration sites as a kind of hypersensitive response [2] and the defense also includes ethylene production, ROS-burst [3], and other plant immune reactions known from PTI (PAMP-triggered immunity). To enable tomato for the recognition of *C. reflexa*, two components are important: A perception system in tomato and a pathogen-associated molecular pattern (PAMP) from *C. reflexa*. First, Cuscuta Receptor 1 (CuRe1), which initiates PTI-responses, has been identified in tomato [3]. CuRe1 detects the *Cuscuta-*derived molecular pattern, a *C. reflexa* glycine-rich protein (CrGRP) that occurs in all tissues of giant dodder [3,4]. Glycine-rich proteins (GRPs) belong to a protein superfamily which all share a number of semi-repetitive glycine-rich motifs. Otherwise, they can be divided into five main subclasses, all showing different functions in distinct tissues and developmental stages [5]. In some plants, GRPs show differentially regulated expression modulated by abiotic stresses like osmotic stress [6] and temperature stress [7]. In other plants, GRP gene modulation by pathogens led to the discovery of antiviral [8], antibacterial [9] and antimicrobial [10] activities (overview in Figure 2). However, in general, not much is known about the physiological or molecular function of GRPs in plants.

**Figure 2.**
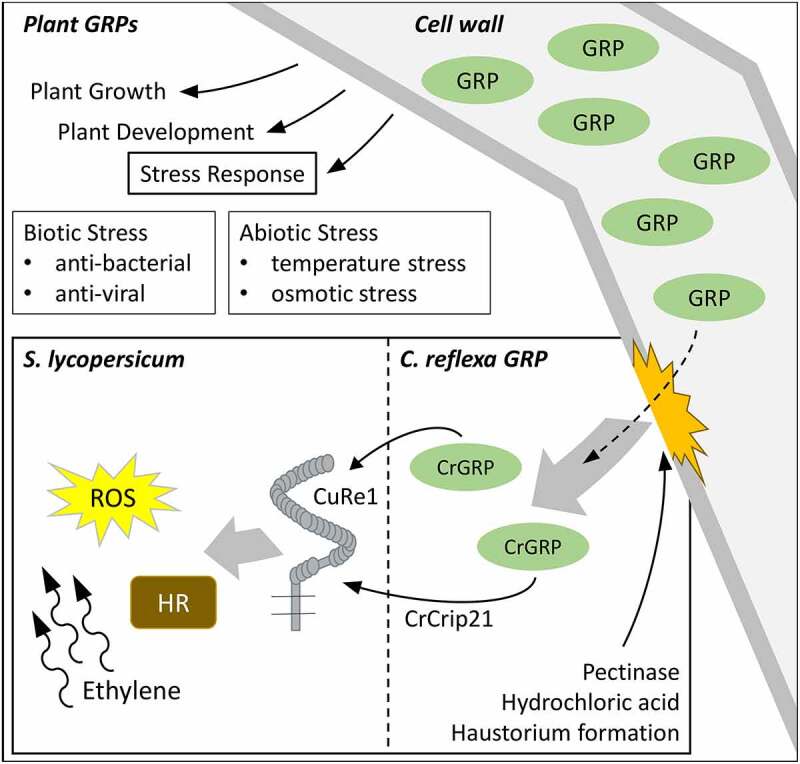
Roles of GRPs in plants and how CrGRP can initiate host defense during haustorium formation. GRPs belong to a superfamily of proteins that are able to fulfill multiple roles in plant growth, plant development and stress responses. In *Cuscuta*-host interactions, the cell wall derived CrGRP, or its minimal motif CrCrip21, is released naturally during host invasion or by pectinase and hydrochloric acid treatment. CrGRP/CrCrip21 is recognized by tomato CuRe1 and initiates defense responses like hypersensitive response (HR), reactive oxygen species (ROS) – and ethylene-production

The CrGRP that is detected by tomato CuRe1 induces immune responses and belongs to the class II of the GRP superfamily. The 116 aa long protein possesses an additional c-terminal cysteine-rich region – typical for class II GRPs – and an n-terminal signal sequence. The signal sequence predicts an extracellular localization of the CrGRP. As a minimal peptide epitope that is sufficient to be detected by CuRe1, a 21 aa long peptide sequence in the c-terminal region, Cysteine-rich peptide 21 (Crip21), has been elucidated. Since GRP studies indicate a structural function [11] as scaffold or agglutinating agents for cell wall stability [12], the hypothesis arose that the protein or its minimal motif CrCrip21 is freed from the *C. reflexa* cell wall during haustoria penetration and gets recognized through *S. lycopersicums* CuRe1. Indeed, the release of CrGRP from cell wall preparations with pectinase or through rough treatment of whole *C. reflexa* stems with hydrochloric acid and a subsequent application of the obtained extracts to tomato could induce defense-related ethylene responses [4, overview in Figure 2]. Additionally, a co-localization could be visualized between CrGRP-tagRFP and the autofluorescence of cell walls in transiently transformed *Nicothiana benthamiana*. Therefore, the tagRFP fluorescence was excited with a 561 nm laser and emission was measured in a narrow spectrum of 563 nm to 607 nm [13] to exclude chloroplasts autofluorescence. The natural autofluorescence of lignin as a cell wall component was excited in the UV range (405 nm) and the emission was detected between 410 nm and 466 nm [14] (see Figure 1b). A closer analysis of the gray scale spectra of both fluorescence pictures revealed a perfect overlap of CrGRP-tagRFP and cell wall autofluorescence, clearly indicating for a localization of CrGRP to the plant cell wall (see Figure 1c). Gray scale analysis of the fluorescence spectra of several independently transformed *N. benthamiana* (n = 7) confirmed these results.

Besides the function of CrGRP as PAMP being recognized by tomato CuRe1, other physiological roles of GRPs in plants remain unclear and have not yet been described in detail. Although a lot of data concerning different GRPs indicate a variety of functions. The class I GRP PvGRP1.8 from the French bean is, similar to the CrGRP, located at the cell wall and is thought to have functions in protoxylem development as repair system and in stability by connecting the lignin rings of the cell wall [15]. Another class II GRP in *Arbidopsis thaliana* was shown to have a role during wounding and recognition of oligogalacturonide, a damage associated molecular pattern (DAMP). The AtGRP3 is involved in the secondary immune response phase by helping to return to the baseline of the plant immune system [16].

A recent study by Hegenauer et al. 4, uncovers a GRP of dodder as a trigger for host immune responses in a similar manner as it is known for PTI. The role of PAMP, however, is most likely not the prior function of CrGRPs since the protein is highly abundant in all parts and tissues of the *Cuscuta* shoots [3]. The reason for the conservation of the CrGRP, even though its treacherous nature, may be, besides an essential function, the broad host spectrum of *Cuscuta* and consequently the freedom of host choice. Therefore, the original or additional roles of CrGRP in *C. reflexa* remain to be discovered.
